# Matching-adjusted indirect comparison of CPX- 351 in secondary Acute Myeloid Leukemia between the registrative trial and a real-life study

**DOI:** 10.1007/s00277-025-06381-3

**Published:** 2025-05-24

**Authors:** Luana Fianchi, Alfonso Piciocchi, Fabio Guolo, Francesco Marchesi, Giovanni Marsili, Chiara Cattaneo, Michele Gottardi, Francesco Restuccia, Anna Candoni, Elettra Ortu La Barbera, Rita Fazzi, Crescenza Pasciolla, Olimpia Finizio, Nicola Fracchiolla, Mario Delia, Federica Lessi, Michela Dargenio, Valentina Bonuomo, Maria Ilaria Del Principe, Patrizia Zappasodi, Marco Picardi, Claudia Basilico, Monica Piedimonte, Paola Minetto, Patrizia Chiusolo, Lucia Prezioso, Caterina Buquicchio, Lorella Melillo, Daniele Zama, Francesca Farina, Valentina Mancini, Michela Rondoni, Alessandro Busca, Livio Pagano

**Affiliations:** 1 Dipartimento Di Diagnostica Per Immagini, Fondazione Policlinico Universitario A. Gemelli, IRCCS- Università Cattolica del Sacro Cuore, Radioterapia Oncologica Ed Ematologia, Rome, Italy; 2 Unità Di Biostatistica Fondazione Gimema, Rome, Italy; 3 IRCCS Ospedale Policlinico San Martino, Genoa, Italy; 4 IRCCS Istituto Nazionale Tumori Regina Elena, Rome, Italy; 5 SC Ematologia E Dipartimento Di Oncologia Clinica, A.O. Spedali Civili, Brescia, Italy; 6 U.O.C. Oncoematologia Dipartimento Di Oncologia Istituto Oncologico Veneto (IOV)-IRCCS, Castelfranco Veneto, Italy; 7 UOC Ematologia Ospedale Civile, Pescara, Italy; 8 Department of Medical and Surgical Sciences, Azienda Ospedaliera Universitaria Di Modena, Università Di Modena E Reggio Emilia, Modena, Italy; 9 UOC Ematologia Con Trapianto Santa Maria Goretti, Latina, Italy; 10 Hematology Unit – A.O.U.P. Ospedale Santa Chiara, Pisa, Italy; 11 Haematology Unit, IRCCS Istituto Tumori “Giovanni Paolo II”, Bari, Italy; 12 AORN Cardarelli, Naples, Italy; 13 Fondazione IRCCS Cà Granda-Ospedale Maggiore Policlinico Di Milano, Milan, Italy; 14 Azienda Ospedaliero-Universitaria Consorziale Policlinico Di Bari, Bari, Italy; 15 Ematologia E Immunologia, Clinica Azienda Ospedaliera Di Padova, Padua, Italy; 16 UO Ematologia E Trapianto CSE V.Fazzi, Lecce, Italy; 17 Dipartimento Di Scienze Cliniche E Biologiche, Università Di Torino, Turin, Italy; 18 Dipartimento Di Biomedicina E Prevenzione, Università Degli Studi Di Roma “Tor Vergata”, Rome, Italy; 19 Fondazione IRCCS Policlinico San Matteo, Pavia, Italy; 20 Ematologia, AOU Federico II, Naples, Italy; 21 Azienda Socio Sanitaria Territoriale Dei Sette Laghi, Varese, Italy; 22 AOU Sant’Andrea, Rome, Italy; 23 Ematologia E Trapianto, IRCCS Ospedale Policlinico San Martino, Genoa, Italy; 24 Ematologia, Ospedale Di Parma, Parma, Italy; 25 Sc Ematologia Con Trapianto, Ospedale Dimiccoli, Barletta, Italy; 26 Ematologia, Policlinico OU, Foggia, Italy; 27 Policlinico Sant’Orsola Malpighi, Bologna, Italy; 28 Dipartimento Di Oncologia, U.O. Ematologia E Trapianto Midollo, Istituto Scientifico San Raffaele, Milan, Italy; 29 Divisione Di Ematologia, Ospedale Niguarda, Milan, Italy; 30 U.O.C. Di Ematologia, Azienda Unità Sanitaria Locale Della Romagna, Ravenna, Italy; 31 SC Ematologia, Ospedale, AOU Città Della Salute E Della Scienza, Turin, Italy

**Keywords:** Secondary acute myeloid leukemia, CPX- 351, Matching-adjusted indirect comparison

## Abstract

A real-life study on CPX-351 and the standard arm (‘7 + 3’) of the CPX-351 registrative trial in adults with secondary Acute Myeloid Leukemia were compared by an unanchored Matching-adjusted indirect comparison (MAIC), in order to evaluate the efficacy and toxicity of CPX-351. Results of this study are important to confirm the role of CPX-351 in significantly improving survival and remission rates compared with ‘7 + 3’ with a good safety profile in AML patients with high-risk features, a target group traditionally with a very poor prognosis. Moreover, this pilot analysis underlines the potentiality of the statistical method to compare studies with strong differences.

## Introduction

Approximately a 25% of the total cases of acute myeloid leukemia (AML) are subsequent to previous hematological disorders (sAML) or developing after chemotherapy or radiotherapy (tAML) [1]. Furthermore, AML with myelodysplasia-related changes (MRC-AML) is defined by the history of a myelodysplastic syndrome (MDS), signs of MDS-related cytogenetic abnormalities and/or dysplasia [2]. All the above occur more frequently with advancing age and are associated with adverse genetics and multidrug resistance phenotype ([3, 4].

In the last 40 years, the ‘7 + 3’ regimens have been a standard for AML induction therapy (5). In older adults and patients with secondary AML, ‘7 + 3’ induction is associated with lower remission rates, increased early mortality, and higher relapse rates than in younger adults and patients with de novo AML [5].

CPX- 351 is a liposomal encapsulation of cytarabine and daunorubicin in a 5-to- 1 molar ratio. The registration study with a trial comparing the classic ‘7 + 3’ Vs. CPX- 351 has demonstrated the greater efficacy in the treatment of sAML [5, 6].

CPX- 351 has received Food and Drug Administration (FDA) in 2017 and European Medicine Agency (EMA) in 2018 approval for the treatment of adult patients with tAMLs, sAMLs, or MRC–AML.

After the registration of CPX- 351, several real-life studies have been performed to evaluate the efficacy and safety of this new formulation, and recently an Italian multicenter study demonstrated the low rate of infections and treatment-related mortality among 200 patients]7].

In this analysis, a real-life study on the use of CPX- 351 and the standard arm (‘7 + 3’) of the CPX- 351 registrative trial in adults with sAML were compared by an unanchored Matching-adjusted indirect comparison (MAIC), in order to evaluate the efficacy and toxicity of CPX- 351.

## Methods and patients

MAIC methodology use individual patient data (IPD) from trials of one treatment to match baseline summary statistics reported from trials of another treatment, while indirect comparisons based only on aggregate data can be limited by cross-trial differences in patient populations, differences in the definitions of outcome measures, and sensitivity to modelling assumptions. By combining IPD with published aggregate data, MAIC can reduce observed cross-trial differences that can be potentially either prognostic or treatment effect modifiers and provide decision makers with timely comparative evidence [8].

This analysis aimed to test the feasibility to compare individual patients’ data with aggregated published results and evaluate the rate of infections of CPX- 351 in real life vs the ‘7 + 3’ regimen and their impact on the survival outcomes.

Patients-level data from GIMEMA-SEIFEM real-life study on the use of CPX- 351, including all consecutive patients with AML from 30 Italian hematologic centers who received at least 1 course of CPX- 351 from July 2018 to June 2021 according to clinical practice (n = 200) was weighted for the aggregated patients’ characteristics from the standard arm of the CPX- 351 trial (‘7 + 3’ arm, n = 156). The study was approved by the Ethics Committee of the coordinating center, Fondazione Policlinico Universitario Agostino Gemelli—IRCCS, Università Cattolica del Sacro Cuore of Rome, Italy (Study ID: 3405), and by the respective ethics committees of all participating centers. Written informed consent for data collection was obtained from each patient enrolled. The study was conducted according to the Declaration of Helsinki.

Accordingly, weighted Overall Survival and Event-Free Survival (w–OS, w-EFS) estimates, as well as rates of febrile neutropenia, fever of unknown origin (FUO), pneumonia, complete recovery (CR), and the interval of polymorphonuclear neutrophil (PMN) recovery, were computed.

## Results

Five potential effect modifiers were identified and used for adjustment: age, sex, AML subtype (tAML, sAML, MDR), prior hypomethylating agents (HMA) exposure and transplant.

The study included 200 patients treated with CPX- 351 in a real-life setting across 30 Italian hematologic centers.

Table 1 describes these characteristics in the original real-life study and adjusted.

**Table 1:** Potential effect modifiers identified and used for adjustment: age, sex, AML subtype (tAML, sAML, MDR), prior hypomethylating agents (HMA) exposure and transplant in the original real-life study and adjusted

	GIMEMA-SEIFEM CPX- 351 real-life study	CPX- 351 trial (‘7 + 3’ arm)	Adjusted GIMEMA-SEIFEM CPX- 351 real-life study
Age (median)	65yy	67yy	67yy
Sex M/F	1.04	1.6	1.6
AML subtype (tAML)	26%	21%	22%
AML subtype (sAML)	35%	54%	54%
AML subtype (MDR)	39%	24%	25%
Prior HMA	20%	46%	46%
Transplant	47%	25%	25%

The median age of the cohort was 65 years, with a range spanning from 18 to 80 years. The gender distribution was balanced, with 51% of patients being male. Regarding disease classification, 26% of patients were diagnosed with therapy-related acute myeloid leukemia (tAML), 35% with secondary AML (sAML), and 39% with AML with myelodysplasia-related changes (MDR-AML). A history of prior exposure to hypomethylating agents (HMA) was documented in 20% of cases. A significant proportion of patients, amounting to 47%, underwent hematopoietic stem cell transplantation (HSCT) as part of their treatment.

To ensure comparability between the real-world CPX- 351 cohort and the ‘7 + 3’ standard arm of the registrative trial, a Matching-Adjusted Indirect Comparison (MAIC) was performed. The adjustment process resulted in a more balanced patient distribution, aligning baseline characteristics between the two groups. After weighting, the median age increased from 65 to 67 years, while the percentage of patients with sAML rose from 35 to 54%. The proportion of patients with prior HMA exposure increased from 20 to 46%, and the rate of HSCT decreased from 47 to 25%, reflecting a population more similar to that of the registrative trial.

In terms of treatment response, the unadjusted rate of complete remission (CR) following CPX- 351 treatment in the real-world cohort was 65%, which decreased to 55% after weighting. In contrast, the CR rate in the ‘7 + 3’ arm of the registrative trial was 33.3%, demonstrating a higher response rate with CPX- 351. The adjusted early mortality rate at 30 days and at 60 days was 3.8% and 11% respectively, compared to those observed in the registrative trial, where early mortality rates with CPX- 351 and ‘7 + 3’ were 5.9% and 10.6% at day 30, and 13.7% and 21.2% at day 60, respectively.

**Fig. 1:**
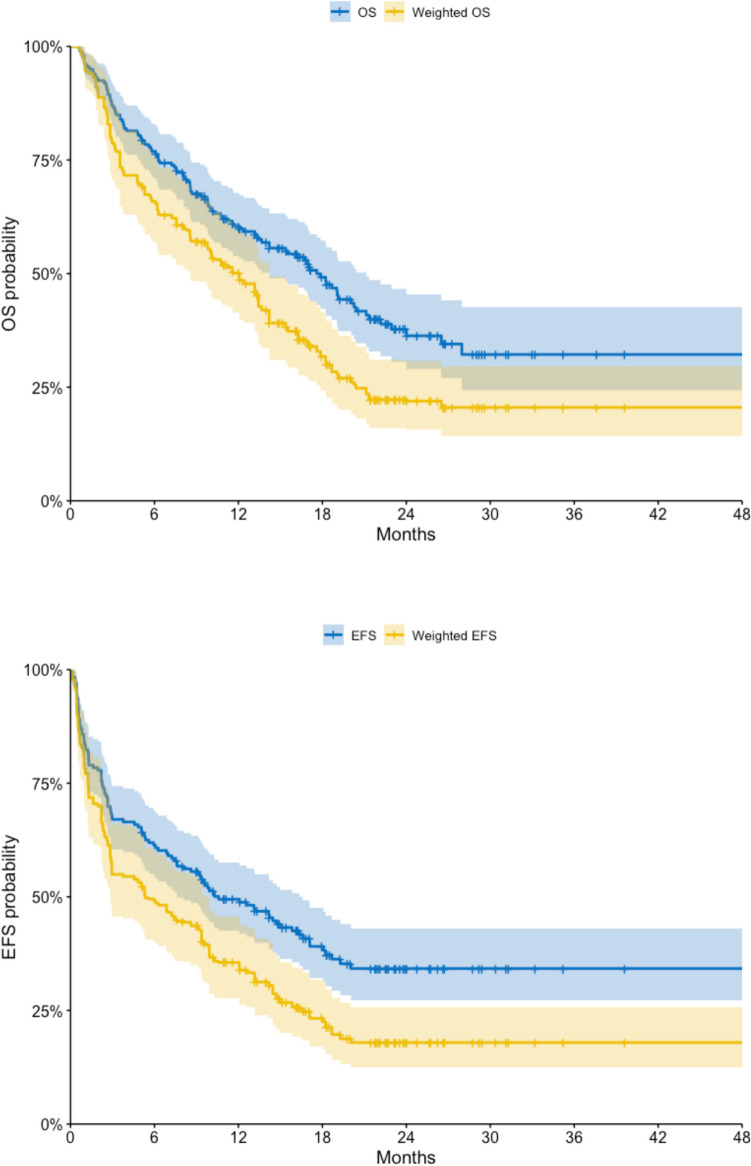
Kaplan–Meier curves for overall survival (OS) and event-free survival (EFS) in the real-life CPX- 351 study (unweighted) versus its weighted estimates

Survival outcomes were also analyzed pre- and post-weighting **(**Fig. 1**)**. The observed median overall survival (OS) in the CPX- 351 real-world cohort was 18 months, with a 95% confidence interval of 14 to 21 months. Following MAIC adjustment, the weighted median OS was 12 months, ranging from 8.5 to 14 months. In comparison, the median OS in the ‘7 + 3’ arm of the registrative trial was notably lower, at 5.9 months (95% CI: 5.0–7.7). Similarly, the median event-free survival (EFS) in the real-world CPX- 351 cohort was 11 months (95% CI: 7.6–17), while the weighted EFS was reduced to 5.4 months (95% CI: 2.8–9.8). The EFS in the ‘7 + 3’ cohort remained markedly inferior at 1.3 months (95% CI: 1.0–1.6).

The study also evaluated the hematologic recovery profile and the incidence of infectious complications. The median time to polymorphonuclear neutrophil (PMN) recovery in the CPX- 351 real-world cohort was 19 days, with a weighted median of 17.8 days, indicating a prolonged period of neutropenia but one that remained within the expected range for CPX- 351 treatment. Febrile neutropenia (FN), defined according to Infectious Diseases Society of America (IDSA) criteria, grade 1–4, was a frequent adverse event, with an incidence of 74% in the real-world setting, which increased slightly to 76% after MAIC adjustment. In the ‘7 + 3’ arm of the registrative trial, the FN rate was reported at 70.9%. The incidence of fever of unknown origin (FUO) in CPX- 351-treated patients was 37% before weighting and 33% post-weighting, while pneumonia was recorded in 12% of patients before weighting and 15% post-weighting.

## Discussion

In vitro studies showed that CPX- 351 enhances drug synergy and extends the half-life of both drugs, improving their bone marrow penetration and accumulation [9, 10]. However, its pharmacokinetics lead to prolonged post-chemotherapy cytopenia, with neutropenia recovery around 36 days (vs. 32 days with traditional chemotherapy) [5].

In the phase II trial, nonhematologic common grade 3/4 adverse events included febrile neutropenia (34%), pneumonia (23%), and sepsis (16%) [11]. In the randomized phase III trial, febrile neutropenia was the most frequent adverse event (68.0% vs. 70.9% in ‘7 + 3’), with infection-related grade 3 to 5 events occurring in 83.7% of CPX- 351 patients vs. 86.1% in ‘7 + 3’ [5]. In a French cohort, 91% had febrile neutropenia, 36% had pulmonary infections, and 10% were treated for invasive pulmonary aspergillosis [12]. In an early access program for older patients, febrile neutropenia (31%) and infections (6%) were the most common treatment-emergent adverse events [13].

The SEIFEM study confirmed CPX- 351’s safety profile in real-world settings, with infectious complications similar to pivotal trials. Despite prolonged neutropenia, fungal infections were low (5.5%) and infection-related mortality was 6% [7].

The MAIC method in this study allowed for a robust comparison of two clinical trials in AML treatment, demonstrating its potential for comparing studies with large differences in patient selection. Baseline characteristics showed no significant sex distribution differences between groups. The GIMEMA-SEIFEM cohort was slightly younger, with different secondary AML subtypes. Notably, fewer patients (20%) in the real-life study had prior hypomethylating agents compared to the adjusted group (46%), and more patients (47%) in the real-life study underwent transplants compared to the post-weighted group (25%). This disparity likely impacts survival outcomes, reflecting probably a greater real-world experience with the drug.

Survival outcomes in the real-life cohort were better than the registration trial’s median values, even after adjustment. In this MAIC analysis, CPX- 351 showed higher OS and EFS compared to ‘7 + 3’. Safety data indicated similar recovery times for PMN and comparable infectious outcomes, with febrile neutropenia being the main difference. Pneumonia risk was lower in the CPX- 351 group than in the ‘7 + 3’ arm.

Ultimately the data obtained by this MAIC gives us back the profile of an easy-to-handle drug; this can be considered of utmost importance, because the target population is mainly a fragile population, often with a history of a previous neoplasm or myelodysplasia which leads to an increased risk of complications.

The Kaplan–Meier Survival curves, even adjusted, show better results of CPX- 351 therapy compared to conventional cytarabine plus daunorubicin regimen.

In conclusion, results of this study are important to confirm the role of CPX- 351 in significantly improving survival and remission rates compared with ‘7 + 3’ with a good safety profile in AML patients with high-risk features, as sAML or therapy-related AML, a target group traditionally with a very poor prognosis.

## Data Availability

Data sharing will be available upon request to the Corresponding Author.
